# Utilization of Dimethyl Fumarate and Related Molecules for Treatment of Multiple Sclerosis, Cancer, and Other Diseases

**DOI:** 10.3389/fimmu.2016.00278

**Published:** 2016-07-22

**Authors:** Zaidoon Al-Jaderi, Azzam A. Maghazachi

**Affiliations:** ^1^Department of Clinical Sciences, College of Medicine and Sahrjah Institute for Medical Research, University of Sharjah, Sharjah, United Arab Emirates

**Keywords:** NK cells, dimethyl fumarate, cancer, multiple sclerosis, monomethyl fumarate

## Abstract

Several drugs have been approved for treatment of multiple sclerosis (MS). Dimethyl fumarate (DMF) is utilized as an oral drug to treat this disease and is proven to be potent with less side effects than several other drugs. On the other hand, monomethyl fumarate (MMF), a related compound, has not been examined in greater details although it has the potential as a therapeutic drug for MS and other diseases. The mechanism of action of DMF or MMF is related to their ability to enhance the antioxidant pathways and to inhibit reactive oxygen species. However, other mechanisms have also been described, which include effects on monocytes, dendritic cells, T cells, and natural killer cells. It is also reported that DMF might be useful for treating psoriasis, asthma, aggressive breast cancers, hematopoeitic tumors, inflammatory bowel disease, intracerebral hemorrhage, osteoarthritis, chronic pancreatitis, and retinal ischemia. In this article, we will touch on some of these diseases with an emphasis on the effects of DMF and MMF on various immune cells.

## Introduction

Twenty years ago, there was no treatment for multiple sclerosis (MS). Today, there are wide varieties of immunomodulatory drugs, which have been licensed to treat MS patients. For relapsing-remitting MS (RRMS) patients, several drugs have been approved. During the 1990s, beta interferon and glatiramer acetate were the only drugs available. Other drugs, such as natalizumab, teriflunomide, and fingolimod, were later approved. These drugs modify the immune system to slow disease progression, decrease attacks, and reduce the development of new brain lesions.

Dimethyl fumarate (DMF) has been lately approved by the US Food and Drug Administration (FDA) as an oral drug for MS patients. This drug was first used to treat inflammatory skin diseases, such as psoriasis. The beneficial effects of this medication corroborated with regulating CD4^+^ Th1 cell differentiation. In clinical trials, it showed positive benefits for MS patients by lowering risk of relapse and reducing the number of brain lesions (1–6).

**Figure 1:**
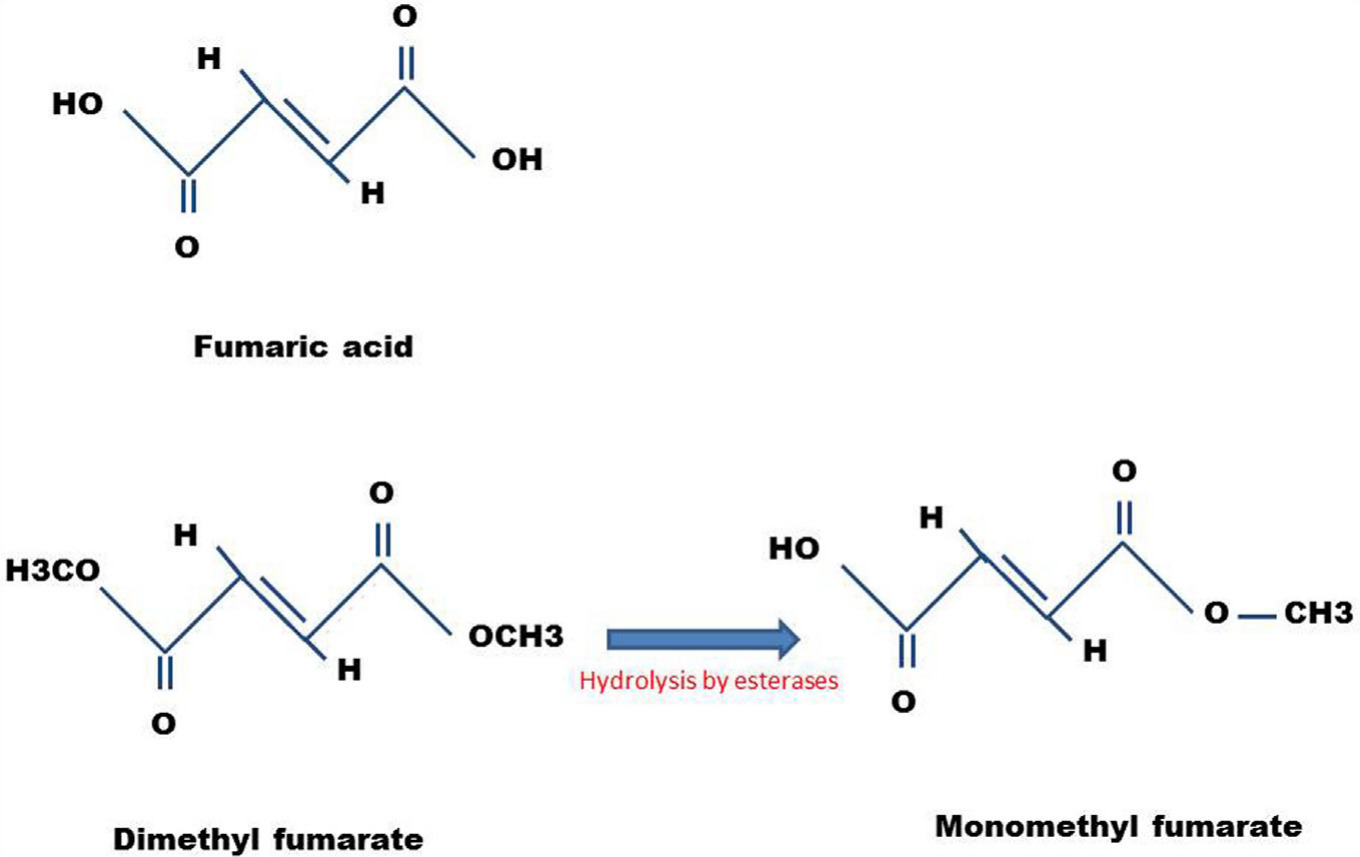
**Chemical structures of DMF and MMF**. Also shown is the structure of fumaric acid, the precursor molecule.

The mechanism of action is not fully known. After oral intake, DMF is completely absorbed in the small intestine, and only small amounts are excreted in the feces and urine (7). DMF possesses a short half-life of ~12 min inside the body (8). After absorption, DMF is rapidly hydrolyzed by esterases to monomethyl fumarate (MMF) (9), which has a short half-life of 36 h. This molecule interacts with the immune cells in the blood circulation and crosses the blood–brain barrier (BBB) to the central nervous system (CNS) (10). Figure 1 shows the structures of DMF and MMF.

Dimethyl fumarate is an α, β-unsaturated carboxylic acid ester. It is demonstrated that DMF by activating nuclear factor erythroid 2-related factor (Nrf2), stimulated the production of glutathione (GSH), the cells most important scavenger of reactive oxygen species (ROS) (11), hence, protecting against ROS-induced cytotoxicity. Further studies demonstrate that DMF downregulated nuclear factor kappa B (NF-κB) in cells, inhibited the anti-apoptotic protein Bcl-2, and induced apoptosis. Cells challenged with oxidative stressors increase their antioxidant capacity as a response to increase ROS production and maintain homeostasis. Nrf2 acts as a key control of the redox gene transcription; under oxidative stress, the Nrf2 signaling is activated to enhance the expression of a large number of antioxidants and enzymes that restore redox homeostasis. Nrf2 interacts with the cysteine-rich protein Kelch-like ECH-associated protein 1 (Keap1) and acts as an adaptor protein for the Cul3-dependent E3 (Cul3) ubiquitin ligase complex. In normal conditions, Keap1 promotes ubiquitination and repeatedly eliminates Nrf2 within a half-life of 13–21 min (12, 13). Keap1 possesses many cysteine residues in the amino acid terminal that act as sensors detecting changes in cellular redox state. During cellular stress, Keap1 is less effective at promoting Nrf2 degradation (12, 14).

Under normal conditions, Nrf2 is sequestered in the cytoplasm *via* binding to its inhibitory molecule Keap1. ROS/stress causes dissociation of Nrf2-Keap1 complex, leading to activation of Nrf2 and its translocation into the nucleus. In the nucleus, Nrf2 heterodimerizes with other transcription factors, such as MAF, and consequently, binds the antioxidant responsive elements (ARE) in the target genes. Nrf2 promotes transcriptional activation of antioxidants and detoxifying enzymes. At the same time, phosphorylation of the repressor molecule IκB by ROS/stress causes activation of NF-κB, leading to activating gene transcription encoding inflammatory mediators. Studies have shown that Nrf2 and NF-κB pathways have inhibitory influence on one another.

## Effects of DMF on the Innate Immune System

The main components of the innate immune system are epithelial barriers, leukocytes, dendritic cells (DCs), and natural killer (NK) cells. NK cells are large granular lymphocytes that spontaneously lyse target cells and are important for defending against viral infections as well as controlling tumor growth. NK cells have also immunoregulatory role by secretion of cytokines, chemokines, as well as cell-to-cell cross-talk (15). These cells express several activating and inhibitory receptors that detect target cells and control NK cell activity. In human, NK cells are divided by the expression of CD56 molecule into CD56^dim^ and CD56^bright^ subsets (15, 16).

The flow cytometric analysis of peripheral blood immune cells in 41 DMF-treated MS patients shows that these patients had significantly fewer circulating CD8^+^ T cells, CD4^+^ T cells, CD56^dim^ NK cells, CD19^+^ B cells, and plasmacytoid DCs (17). Furthermore, the expression of CXCR3^+^ (a potential marker for Th1) and CCR6^+^ (a potential marker for Th17) was reduced, while the number of regulatory T cells (Treg) was unchanged. Interestingly, DMF did not affect circulating CD56^bright^ NK cells, CD14^+^ monocytes, or myeloid DCs. However, DMF-treated patients had significantly fewer CD56^dim^ NK cells when compared with healthy controls (17). A clinical study of 35 RRMS patients at baseline, 3 months, 6 months, and 12 months after initiation of DMF treatment shows that total leukocyte and lymphocyte counts diminished after 6 months, whereas after 12 months of DMF therapy total T cells counts decreased by 44%, CD8^+^ T cell counts declined by 54.6%, and CD4^+^ T cell counts decreased by 39% (18). CD19^+^ B cell counts were also reduced by 37.5%, and eosinophils counts were decreased by 54%, whereas the percentages of neutrophils, monocytes, basophils, and NK cells were not significantly altered (18).

It has been previously demonstrated that MMF is a potent agonist of hydroxycarboxylic acid receptor 2 “(HCA_2_), a G protein coupled receptor also known as GPR109A” (19). It is reported that HCA_2_ mediates the therapeutic effects of DMF or MMF in experimental autoimmune encephalomyelitis (EAE) mouse model (20). Recently, we observed that MMF enhanced primary non-activated human CD56^+^ NK cell lysis of leukemic cell line K562 and B-cell lymphoma RAJI cells *in vitro* (21). Furthermore, MMF upregulated NKp46 expression on the surface of CD56^+^ NK cells, an activity correlated with upregulation of CD107a expression and the release of Granzyme B from CD56^+^ NK cells (21).

Moreover, MMF delayed EAE clinical score in SJL/J mice and prevented the disease progression in treated mice. These results are linked to enhanced NK cells lysis of DCs isolated from the same mice (22). To correlate these findings with human settings, we recently observed that human NK cells incubated with various concentrations of DMF or MMF robustly lysed immature DCs *in vitro* (manuscript in preparation). These findings suggest that one mechanism of action for these “drugs” is plausibly due to activating NK cells to lyse DCs, and consequently, impeding antigen presentation to autoreactive T cells.

Dendritic cells represent key links between the innate and adaptive immune system (23). T cells and NK cells are stimulated through direct contact with activated DCs (24). DCs play a major role in regulating the immune response by releasing cytokines and expressing co-stimulatory molecules. They are capable of processing both exogenous and endogenous antigens and present them in the context of MHC class I or II molecules. It has been reported that DMF inhibited DCs maturation through a reduction in the release of the inflammatory cytokines IL-6 and IL-12. Furthermore, DMF activated type II DCs, which have anti-inflammatory effects and suppressed type I DCs, which are inflammatory (25, 26). DMF induced type II DCs by regulating GSH depletion, followed by increased heme oxygenase-1 (HO-1) expression and suppressing STAT1 phosphorylation in DCs (26). This, combined with the reduction in the inflammatory cytokines by nuclear translocation of NF-κB, resulted in inhibiting CD1a, CD40, CD80, CD86, and HLA-DR expression (27). Consequently, the capacity of DCs to stimulate allogeneic Th1 and Th17 cells is reduced. It was also determined that increased production of the Th2 cytokines and increased expression of IL-10, instead of IL-12 and IL-23 by DCs, enhanced the development of T regulatory (Treg) cells (28).

We reported that monocyte-derived DCs isolated from EAE mice treated with MMF did not increase the expression of CD80 molecule (22). Interestingly, E-cadherin expression was upregulated in EAE mice, and MMF reversed this upregulation. Increased E-cadherin expression suggests a shifting of the immune system toward inflammatory Th1/Th17 response. These results support previous study showing that inflammatory E-cadherin^+^ bone marrow-derived DCs isolated from animals with colitis promoted Th17 response (29). The study also demonstrates that E-cadherin^+^ DCs enhanced Th1 cell responses (29). Furthermore, E-cadherin^+^ DCs increased the number of IFN-γ^+^ CD4^+^ T cells and decreased the number of IL-4^+^ CD4^+^ T cells (30). MMF by decreasing E-cadherin expression on DCs may decrease inflammatory Th1/Th17 proliferation and may enhance the anti-inflammatory Th2 cells.

Although DMF but not MMF induced apoptosis in iDCs and moderately inhibited the ability of DCs to induce proliferation of allogeneic T cells, it is reported that MMF affected the polarization but not maturation of monocyte-derived DCs, resulting in downregulating Th1 lymphocyte responses (31). *In vitro* study for the effects of MMF on DCs differentiation shows that MMF inhibited monocyte-derived DCs differentiation in response to LPS, resulting in cells that are incapable of appropriately mature to DCs. In addition, MMF did not decrease the capacity of DCs to capture antigens, but MMF/DCs interaction resulted in producing low levels of IL-12, IL-10, and TNF-α, whereas IL-8 production was not altered (32). Consequently, MMF/DCs interaction partially affected IFN-γ production by naive T cells, whereas the production of IL-4 and IL-10 was not influenced by MMF (32). Another study demonstrates that DMF inhibited DCs maturation by reducing the production of the inflammatory cytokines IL-6 and IL-12 as well as the expression of MHC class II, CD80, and CD86 (27). Furthermore, immature DCs activated fewer T cells characterized by low IFN-γ and IL-17 production (27). In contrast, de Jong et al. (33) demonstrate that MMF increased the production of IL-4 and IL-5 without altering the production of IL-2 and interferon-γ in stimulated peripheral blood mononuclear cells challenged with bacterial antigens.

## Treatment of Psoriasis and Other Skin Diseases with DMF

Psoriasis is a type-1 cytokine-mediated chronic autoimmune skin disease aided by the infiltration of Th1/Th17 cells into the skin (34–36). DMF is utilized to treat psoriasis in European countries for more than 30 years. Fumaric acid was first used for treatment of psoriasis by the German chemist Walter Schweckendiek in 1959. In 1994, DMF was licensed in Germany under the trade name Fumaderm for the treatment of psoriasis. DMF inhibited Janus kinas (JAK) signaling and interfered with intracellular proteins trafficking and consequently, inhibited the release of pro-inflammatory cytokines, such as IL-12, IL-23, and TNF, whereas the release of anti-inflammatory cytokines, such as IL-10, was increased. DMF also inhibited the production of IFN-γ and enhanced the production of IL-10 in the culture of psoriatic keratinocytes (37).

Previous experimental and clinical studies were focused on the mechanism of action for DMF that could affect the immune system. The immunohistochemical studies of psoriatic plaques indicate that DMF has several anti-inflammatory effects *via* a number of pathways, leading to reduction in the levels of several inflammatory T cell subsets (38, 39) and decreased recruitment of inflammatory cells (40). The ability of DMF or MMF to induce apoptosis of CD4^+^ and CD8^+^ T cells and *in vitro* switching the immune system toward a Th2 anti-inflammatory type response in psoriasis patients could be through impaired DCs maturation and induction of apoptosis. In addition, DMF inhibited the formation of new blood vessels, a process that is involved in the formation of psoriatic plaques (41).

Clinical studies demonstrate that DMF reduced CD4^+^ T cells and CD8^+^ T cells by inducing apoptotic cell death (42). *In vivo* studies indicate that DMF inhibited T cell mediated organ rejection in a rat model (43). A study of allergic contact dermatitis, a skin disorder in which an exaggerated T cell response occurs, shows that DMF suppressed allergen-induced T cell proliferation, corroborated with modulating cytokines/chemokines expression by reducing the levels of IFN-γ but not IL-5 and downregulating CXCR3 but not CCR4 expression (44).

## Treatment of Multiple Sclerosis Patients with DMF

Multiple sclerosis is a chronic inflammatory autoimmune disease of the CNS in which the insulating myelin sheaths of nerve cell axons in the brain and spinal cord are attacked by the immune system (45). The principal mechanism responsible for this disease is still incompletely understood. The consensus is that activated T cells attack oligodendrocytes, leading to destruction of myelin sheaths (demyelination). Furthermore, the presence of inflammatory T cells in the CNS triggers recruitment of more T cells, B cells, dendritic cells, microglia cells, and NK cells (46). Due to the progressive neurodegenerative nature of MS, therapeutic modalities that exhibit direct neuroprotective effects are needed. A phase 3 clinical trial study of 2667 RRMS patients demonstrates the efficacy and safety of DMF in MS (1). *In vitro* study indicates that DMF increased the frequency of the multipotent neurospheres resulting in the survival of mouse and rat neural stem progenitor cells (NPCs) following oxidative stress with hydrogen peroxide (H_2_O_2_) treatment (47). Using motor neuron survival assay, DMF significantly promoted survival of motor neurons under oxidative stress. Furthermore, DMF increased the expression of Nrf2 at both RNA and protein levels in the NPC cultures (47).

There is agreement that antioxidants reduce the risk of certain pathological conditions, such as neurodegenerative diseases. *In vivo* animal studies have shown that DMF or MMF inhibited the disease course in the EAE model (48). It is also demonstrated that MMF crossed the BBB, indicating it may have a direct cytoprotective function in the CNS (49). The detoxification capabilities of DMF or MMF reduced the production and release of inflammatory molecules, such as TNF-α, IL-1β, and IL-6 as well as nitric oxide from microglia and astrocytes activated with LPS *in vitro* (50, 51). DMF or MMF increased the production of detoxification enzymes, such as nicotinamide adenine dinucleotide phosphate quinone reductase 1 (NQO-1), HO-1, and cellular glutathione, abolishing NF-kB translocation into the nucleus (52). NQO-1 is also detected in the liver and in the CNS of DMF-treated animals. This results in decreased expression of NF-kB-dependent genes that regulate the expression of inflammatory cytokines, chemokines, and adhesion molecules, and consequently, reduced the damage to CNS cells. Reducing the expression of adhesion molecules in the BBB represents a critical step in the transmigration of immune cells into the CNS. DMF inhibited TNF-α-induced expression of intracellular adhesion molecule-1 (ICAM-1), E selection, and the vascular cell adhesions molecule-1 (VCAM-1) in endothelial cells *in vitro* (53, 54). This is correlated with activating Nrf2 (55–58), which is released from the Keap-1complex *via* the activity of fumarates (see above). This may lead to reducing free radicals, preventing the synthesis of reactive nitrogen species, and thus protecting the CNS from degeneration and axonal loss (59, 60). These immunomodulatory activities of DMF or MMF, which constitute inhibiting cytokine production and nitric oxide synthesis, are important for the protection of oligodendrocytes against ROS-induced cytotoxicity and consequently, oligodendrocytes survival during an oxidative attack is augmented.

Multiple sclerosis animal models, such as EAE, are induced by immunization with different myelin antigens, such as proteolipid peptide (PLP_139–151_) in SJL/J mice, an animal model disease that may represent relapsing-remitting form of MS, or C57BL/6J mice immunized with MOG_35–55_, a model closely resembles chronic progressive MS. These models are characterized by inflammation, demyelination, and axonal lose. Treatment of EAE mice with DMF reduced macrophage-induced inflammation in the spinal cord (48). DMF suppressed Th1 and Th17 cell differentiation as well as expression of pro-inflammatory cytokines IFN-γ, TNF-α, and IL-17 (61). The drug also promoted Th2 cells that produce IL-4, IL-5, and IL-10 (33). In chronic MS, microglia cells are activated and released pro-inflammatory cytokines and stress-associated molecules leading to neurodegeneration and alteration of synaptic transmission (62). Modulation of microglia activation toward an alternatively activated phenotype can modify the outcome of some experimental models of neurological diseases. The study on EAE demonstrates that exposure to MMF switched the molecular and functional phenotype of activated microglia from pro-inflammatory type to neuroprotective effect (49). This switch in activity may occur through activation of HCAR2. MMF binding to HCAR2 triggered a pathway driven by the AMPK/Sirt axis resulting in inhibition of NF-κB and reducing pro-inflammatory cytokine production (49).

## Effects of DMF on the Central Nervous System

A recent study reports that administration of DMF protected claudin-5 expression in the BBB along with reduced brain edema formation in C57BL/6 mice undergoing experimental ischemia reperfusion injury (63). Using the immortalized murine brain endothelial cell line bEND.3, a preservation of zonula occludens-1 (ZO-1) and VE-cadherin localization in oxygen–glucose deprived cells in the presence of DMF was observed. Reduced transendothelial migration of the human monocyte cell line THP-1 toward CCL2 chemokine in the lower chamber of a transwell system after pretreatment of the bEND.3 cells with DMF was also noted. Further observations demonstrate decreased ICAM-1, VCAM-1, and E-selectin mRNA expression in bEND.3 cells after treatment with DMF for 6 h (54).

*In vitro* human umbilical vein endothelium examination indicates that DMF or MMF modulated pro-inflammatory intracellular signaling and T-cell adhesiveness of human brain microvascular endothelial cells (64). Neither DMF nor MMF reduced the basal expression of ICAM-1 under inflammatory condition or blocked NF-κB in human brain microvascular endothelial cells compared to solvent control. Hence, it is suggested that brain endothelial cells do not directly mediate a potential blocking effect of fumaric acid esters on the infiltration of inflammatory T cells into the CNS (64). It is also determined that DMF ameliorated inflammation, reduced BBB permeability and improved neurological outcomes by casein kinase 2 and Nrf2 signaling pathways in experimental intracerebral hemorrhage (ICH) mouse model (65).

Evidence from clinical and animal studies suggests that inflammation and oxidative stress, which occur after hematoma formation, are involved in ICH-induced secondary brain injury and neurological dysfunction (66). VCAM-1 and ICAM-1 are adhesion molecules expressed in the endothelium important during inflammation and after tissue injury. Both are increased upon activation of NF-κB-mediated TNF-α signaling pathway. TNF-α increases early onset endothelial adhesion by protein kinase C-dependent upregulation of ICAM-1 expression, which exacerbates ICH. Investigating the experimental autoimmune neuritis indicates that DMF treatment reduced the neurological deficits by ameliorating inflammatory cell infiltration and demyelination of sciatic nerves. In addition, DMF treatment decreased the level of pro-inflammatory M1 macrophages, while increasing the number of anti-inflammatory M2 macrophages in the spleens and sciatic nerves of EAN rats (67, 68). In RAW 264.7 macrophage cell line, a shift in macrophage polarization from M1 to M2 phenotype was demonstrated to be dependent on DMF application. In sciatic nerves, DMF treatment elevated the level of Nrf2 and its target HO-1, which may facilitate macrophage polarization toward M2 type (68). In addition, by reducing NF-kB in astrocytes, DMF inhibited the degradation of IkBa and reduced the expression of nitric oxide synthase (69). Moreover, DMF improved the inflammatory milieu in the spleens of EAN rats, characterized by downregulating mRNA for IFN-γ, TNF-α, IL-6, and IL-17 and upregulating mRNA level for IL-4 and IL-10 (68).

## Effects of DMF on Tumor Development

It has been reported that fumarase is involved in DNA repair (70). By studying yeast cells, it is observed that cytosolic fumarase plays a role in detecting and repairing DNA damage, particularly double-stranded DNA breaks. According to this theory, if the cells lack the fumarase, they may need to repair damaged DNA and are most likely prone to develop tumors. Further study on the role of redox demonstrates that high levels of ROS are harmful to normal cells and may lead to development of tumor by inducing DNA damage. Malignant transformation also increases cellular stress, leading to high ROS levels. On the other hand, Keap1-Nrf2 system protects cells from the effects of oxidants by regulating the expression of cytoprotective proteins (71). *In vivo* evidence indicates that Nrf2 has a protective role against tumor development in mouse models and in prostate cancer in humans (72). The mechanism by which Nrf2 is protective against tumor development has been attributed to the ability of Nrf2 to reduce the amount of ROS and DNA damages in cells.

It has also been demonstrated that DMF inhibited the proliferation of A375 and M24met cell lines and reduced melanoma growth and metastasis in experimental melanoma mouse models (73). Furthermore, DMF arrested the cell cycle at the G2-M boundary and was pro-apoptotic, inhibiting tumor cell growth. On the other hand, MMF increased primary human CD56^+^ NK cell lysis of K562 and RAJI tumor cells, suggesting that this molecule may have *in situ* antitumor activity (21).

## Role of DMF in Gastrointestinal Ulceration

It has been demonstrated that stress can play a pathogenic role for gastrointestinal ulceration, by disrupting gastric mucosal defensive barrier (74). Activators of stress give rise to the release of corticotropin-releasing hormone (CRH). CRH acts on the pituitary gland and stimulates the secretion of ACTH, which promotes glucocorticoids release from the adrenal cortex (75). Glucocorticoids not only interfere with tissue repair, elevate levels of gastric acids, and pepsin but also reduce the secretion of gastric mucus and eventually impair gastric mucosal barrier resulting in peptic ulcer. Low daily oral doses of MMF may prevent the chronic foot-shock stress-induced gastric ulcers and may associate with differential hormonal and oxidative processes (76). MMF suppressed the stress-induced elevation in adrenal gland corticosterone level and modulated the oxidative stress responses. Interestingly, DMF did not inhibit the effect of innate defense against microorganism. Treatment of monocytes and neutrophils with DMF after stimulation with *Staphylococcus aureus, Escherichia coli*, or the yeast *Candida albicans* in addition to zymosan particles or the tripeptide fMLP resulted in increased production of superoxide anion, which exerts anti-microbial effects (77).

## Effects of DMF on Collagen Type II Degradation

*In vivo* study of collagen type II degradation suggests that DMF ameliorated the disease by inhibiting the expression of metalloproteinase (MMP)-1, MMP-3, and MMP-13 that are induced by TNF-α (78). DMF may attenuate MMPs expression by suppressing JAK1 and JAK2/STAT3 pathways and by blocking TNF-α-induced STAT3 phosphorylation and DNA-binding activity (79). *In vivo* mice study on renal fibrosis, where TGF-β plays a key role in the development of the disease, demonstrates that DMF treatment may prevent renal fibrosis *via* Nrf2-mediated suppression of TGF-β signaling (80).

## Conclusion and Future Directions

Dimethyl fumarate was originally used for treatment of psoriasis. Its success in treating RRMS patients led for its approval as an oral drug to treat MS patients. One mechanism of action that our group pursued is that DMF might enhance natural killer cell lysis of dendritic cells, hence, impeding presenting encephalitogen to autoreactive T cells. Further investigations suggest that DMF may also be used in the oncology field due to its ability to suppress the growth of melanoma cells. On the other hand, it appears that related molecules, such as MMF, may have even more potent antitumor activity than DMF. Although the association among DMF and MMF is at present conjectural, it is documented that MMF has robust antitumor activity by activating natural killer cells to kill tumor cells. This new mechanism of action for MMF should provide imputes for investigating this molecule not only as a therapeutic tool for autoimmune diseases but also for cancer and immunodeficient diseases. Table 1 shows the current knowledge regarding the effects of DMF and MMF on various immune cells.

**Table 1:** **Immunoregulatory effects of DMF and/or MMF on various immune cells**.

Cell type	Molecule	Cytokine/other molecules involved	Effect(s)	Reference
T cells	DMF/MMF	↓IFN-γ, ↓TNF-α, ↓IL-17, ↑IL-4, ↑IL-5, ↑IL-10, ↓CXCR3, ↓CCR6	↓Bcl-2, ↑Apoptosis, ↓Th1, ↓Th17, ↑Th2, ↓CD4, ↓CD8, ↑Treg	(17, 18, 27, 32, 42, 44, 61)
B cells	DMF	↑Nrf2→↑GSH→↓ROS, ↓NF-kB	↓Bcl-2, ↑Apoptosis, ↓CD19 B cells	(17, 18)
Monocytes	DMF	↑Nrf2, ↓NF-kB	No effect on cell numbers, ↑Antioxidant response	(18, 77)
DCs	DMF/MMF	↓GSH→↑HO-1, ↓NF-kB, ↓IL-6, ↓IL-12, ↑IL-10, ↓TNF-α↓, E-cadherin	↑Apoptosis, ↓plasmacytoid DCs, ↓DC maturation, ↓type I DCs, ↑type II DCs	(17, 22, 25–27, 31, 32)
NK cells	DMF/MMF	↑NKp46, ↑CD107, ↑Granzyme B	↓CD56^dim^ NK cells, No effect on CD56^bright^ numbers, ↑CD56^bright^ NK cells lysis of tumor cells, ↑Lysis of DCS	(17, 18, 21, 22)
Macrophages	DMF	↑Nrf2, ↓mRNA of IFN-γ, ↓mRNA of TNF-α, ↓mRNA of IL-6, ↓mRNA of IL-17, ↑mRNA of IL-4, ↑mRNA of IL-10	↓M1 macrophages, ↑M2 macrophages	(68)
Neutrophils	DMF/MMF	↓HCA2	↓Number of infiltrating neutrophils	(20)
Keratinocytes	DMF	↓IL-12, ↓IL-23, ↓TNF, ↓IFN-γ, ↑IL-10, ↓IL-6, ↓TGF-α	↓Proliferation of keratinocytes	(37)
Endothelial cells	DMF	↓TNF-α, ↓ICAM-1, ↓VCAM-1, ↓E-selection, ↑Nrf2	↓BBB permeability→↓Immune cell migration	(41, 54, 55, 65)
Microglia	DMF/MMF	↓IL-1, ↓IL-6, ↓TNF-α, ↓NO, ↑Nrf2→↑GSH→↓ROS, ↓NF-kB, ↑NQO-1, ↑HO-1, ↑HCAR2	↑Antioxidant response, switching activated microglia from pro-inflammatory to neuroprotective	(49, 50, 52)
Astrocytes	DMF/MMF	↑Nrf2→↑GSH→↓ROS, ↓NF-kB, ↓IL-1, ↓IL-6, ↓TNF-α, ↓NO	↑Antioxidant response	(50, 52, 58)
Neurons	DMF	↑Nrf2→↑GSH→↓ROS	↓Apoptosis, ↑Neurons survival under oxidative stress	(47, 58)
Tumor cells	DMF	Arrest the cell cycle at G2-M, ↓pro-apoptotic	↓Proliferation of melanoma cells, ↓Proliferation of tumor cells, ↑Apoptosis	(73)

*↓, decreased; ↑, increased*.

## Author Contributions

Both authors contributed to writing this review article.

## Conflict of Interest Statement

The authors declare that the research was conducted in the absence of any commercial or financial relationships that could be construed as a potential conflict of interest.

## References

[1] Gold R Kappos L Arnold DL Bar-Or A Giovannoni G Selmaj K Placebo-controlled phase 3 study of oral BG-12 for relapsing multiple sclerosis. N Engl J Med (2012) 367:1098–107. 10.1056/NEJMoa1114287 22992073

[2] Kappos L Gold R Miller DH MacManus DG Havrdova E Limmroth V Efficacy and safety of oral fumarate in patients with relapsing-remitting multiple sclerosis: a multicentre, randomised, double-blind, placebo-controlled phase IIb study. Lancet (2008) 372:1463–72. 10.1016/S0140-6736(08)61619-0 18970976

[3] Stangel M Linker RA. Dimethyl fumarate (BG-12) for the treatment of multiple sclerosis. Expert Rev Clin Pharmacol (2013) 6:355–62. 10.1586/17512433.2013.811826 23927662

[4] Marziniak M [Multiple sclerosis: new treatment options]. MMW Fortschr Med (2014) 156(Spec No 1):69–73. 10.1007/s15006-014-2549-1 24930351

[5] Moharregh-Khiabani D Linker RA Gold R Stangel M. Fumaric acid and its esters: an emerging treatment for multiple sclerosis. Curr Neuropharmacol (2009) 7:60–4. 10.2174/157015909787602788 19721818 PMC2724664

[6] Linker RA Gold R. Dimethyl fumarate for treatment of multiple sclerosis: mechanism of action, effectiveness, and side effects. Curr Neurol Neurosci Rep (2013) 13:394–8. 10.1007/s11910-013-0394-8 24061646

[7] Werdenberg D Joshi R Wolffram S Merkle HP Langguth P. Presystemic metabolism and intestinal absorption of antipsoriatic fumaric acid esters. Biopharm Drug Dispos (2003) 24:259–73. 10.1002/bdd.364 12973823

[8] Mrowietz U Christophers E Altmeyer P. Treatment of severe psoriasis with fumaric acid esters: scientific background and guidelines for therapeutic use. The German Fumaric Acid Ester Consensus Conference. Br J Dermatol (1999) 141:424–9. 10.1046/j.1365-2133.1999.03034.x 10584060

[9] Nibbering PH Thio B Zomerdijk TP Bezemer AC Beijersbergen RL Van FR. Effects of monomethylfumarate on human granulocytes. J Invest Dermatol (1993) 101:37–42. 10.1111/1523-1747.ep12358715 8392528

[10] Litjens NH Burggraaf J van SE van GC Mattie H Schoemaker RC Pharmacokinetics of oral fumarates in healthy subjects. Br J Clin Pharmacol (2004) 58:429–32. 10.1111/j.1365-2125.2004.02145.x 15373936 PMC1884599

[11] Mrowietz U Asadullah K. Dimethylfumarate for psoriasis: more than a dietary curiosity. Trends Mol Med (2005) 11:43–8. 10.1016/j.molmed.2004.11.003 15649822

[12] Hong F Sekhar KR Freeman ML Liebler DC. Specific patterns of electrophile adduction trigger Keap1 ubiquitination and Nrf2 activation. J Biol Chem (2005) 280:31768–75. 10.1074/jbc.M503346200 15985429

[13] Kobayashi A Kang MI Watai Y Tong KI Shibata T Uchida K Oxidative and electrophilic stresses activate Nrf2 through inhibition of ubiquitination activity of Keap1. Mol Cell Biol (2006) 26:221–9. 10.1128/MCB.26.1.221-229.2006 16354693 PMC1317630

[14] Kobayashi A Kang MI Okawa H Ohtsuji M Zenke Y Chiba T Oxidative stress sensor Keap1 functions as an adaptor for Cul3-based E3 ligase to regulate proteasomal degradation of Nrf2. Mol Cell Biol (2004) 24:7130–9. 10.1128/MCB.24.16.7130-7139.2004 15282312 PMC479737

[15] Maghazachi AA. Insights into seven and single transmembrane-spanning domain receptors and their signaling pathways in human natural killer cells. Pharmacol Rev (2005) 57:339–57. 10.1124/pr.57.3.5 16109839

[16] Maghazachi AA. Role of chemokines in the biology of natural killer cells. Curr Top Microbiol Immunol (2010) 341:37–58. 10.1007/82_2010_20 20369317

[17] Longbrake EE Ramsbottom MJ Cantoni C Ghezzi L Cross AH Piccio L Dimethyl fumarate selectively reduces memory T cells in multiple sclerosis patients. Mult Scler (2015) 22(8):1061–70. 10.1177/1352458515608961 26459150 PMC4829494

[18] Spencer CM Crabtree-Hartman EC Lehmann-Horn K Cree BA Zamvil SS. Reduction of CD8(+) T lymphocytes in multiple sclerosis patients treated with dimethyl fumarate. Neurol Neuroimmunol Neuroinflamm (2015) 2:e76. 10.1212/NXI.0000000000000076 25738172 PMC4335821

[19] Tang H Lu JY Zheng X Yang Y Reagan JD. The psoriasis drug monomethyl fumarate is a potent nicotinic acid receptor agonist. Biochem Biophys Res Commun (2008) 375:562–5. 10.1016/j.bbrc.2008.08.041 18722346

[20] Chen H Assmann JC Krenz A Rahman M Grimm M Karsten CM Hydroxycarboxylic acid receptor 2 mediates dimethyl fumarate’s protective effect in EAE. J Clin Invest (2014) 124:2188–92. 10.1172/JCI72151 24691444 PMC4001545

[21] Vego H Sand KL Hoglund RA Fallang LE Gundersen G Holmoy T Monomethyl fumarate augments NK cell lysis of tumor cells through degranulation and the upregulation of NKp46 and CD107a. Cell Mol Immunol (2016) 13:57–64. 10.1038/cmi.2014.114 25435072 PMC4711674

[22] Al-Jaderi Z Maghazachi AA Vitamin D_3_ and monomethyl fumarate enhance natural killer cell lysis of dendritic cells and ameliorate the clinical score in mice suffering from experimental autoimmune encephalomyelitis. Toxins (Basel) (2015) 7:4730–44. 10.3390/toxins7114730 26580651 PMC4663530

[23] Steinman RM. The dendritic cell system and its role in immunogenicity. Annu Rev Immunol (1991) 9:271–96. 10.1146/annurev.iy.09.040191.001415 1910679

[24] Fernandez NC Lozier A Flament C Ricciardi-Castagnoli P Bellet D Suter M Dendritic cells directly trigger NK cell functions: cross-talk relevant in innate anti-tumor immune responses in vivo. Nat Med (1999) 5:405–11. 10.1038/7403 10202929

[25] Ghoreschi K Bruck J Kellerer C Deng C Peng H Rothfuss O Fumarates improve psoriasis and multiple sclerosis by inducing type II dendritic cells. J Exp Med (2011) 208:2291–303. 10.1084/jem.20100977 21987655 PMC3201195

[26] Zhu K Mrowietz U. Inhibition of dendritic cell differentiation by fumaric acid esters. J Invest Dermatol (2001) 116:203–8. 10.1046/j.1523-1747.2001.01159.x 11179994

[27] Peng H Guerau-de-Arellano M Mehta VB Yang Y Huss DJ Papenfuss TL Dimethyl fumarate inhibits dendritic cell maturation via nuclear factor kappa B (NF-kappa B) and extracellular signal-regulated kinase 1 and 2 (ERK1/2) and mitogen stress-activated kinase 1 (MSK1) signaling. J Biol Chem (2012) 287:28017–26. 10.1074/jbc.M112.383380 22733812 PMC3431702

[28] Moore KW de Waal MR Coffman RL O’Garra A. Interleukin-10 and the interleukin-10 receptor. Annu Rev Immunol (2001) 19:683–765. 10.1146/annurev.immunol.19.1.683 11244051

[29] Siddiqui KR Laffont S Powrie F E-cadherin marks a subset of inflammatory dendritic cells that promote T cell-mediated colitis. Immunity (2010) 32:557–67. 10.1016/j.immuni.2010.03.017 20399121 PMC2938478

[30] Zhang Y Hu X Hu Y Teng K Zhang K Zheng Y Anti-CD40-induced inflammatory E-cadherin+ dendritic cells enhance T cell responses and antitumour immunity in murine Lewis lung carcinoma. J Exp Clin Cancer Res (2015) 34:11. 10.1186/s13046-015-0126-9 25651850 PMC4323023

[31] Litjens NH Rademaker M Ravensbergen B Rea D van der Plas MJ Thio B Monomethylfumarate affects polarization of monocyte-derived dendritic cells resulting in down-regulated Th1 lymphocyte responses. Eur J Immunol (2004) 34:565–75. 10.1002/eji.200324174 14768062

[32] Litjens NH Rademaker M Ravensbergen B Thio HB van Dissel JT Nibbering PH. Effects of monomethylfumarate on dendritic cell differentiation. Br J Dermatol (2006) 154:211–7. 10.1111/j.1365-2133.2005.07002.x 16433787

[33] de Jong R Bezemer AC Zomerdijk TP Pouw-Kraan T Ottenhoff TH Nibbering PH. Selective stimulation of T helper 2 cytokine responses by the anti-psoriasis agent monomethylfumarate. Eur J Immunol (1996) 26:2067–74. 10.1002/eji.1830260916 8814248

[34] Nestle FO Turka LA Nickoloff BJ. Characterization of dermal dendritic cells in psoriasis. Autostimulation of T lymphocytes and induction of Th1 type cytokines. J Clin Invest (1994) 94:202–9. 10.1172/JCI117308 8040262 PMC296298

[35] Gudjonsson JE Johnston A Sigmundsdottir H Valdimarsson H Immunopathogenic mechanisms in psoriasis. Clin Exp Immunol (2004) 135:1–8. 10.1111/j.1365-2249.2004.02310.x 14678257 PMC1808928

[36] Valdimarsson H Bake BS Jonsdotdr I Fry L. Psoriasis: a disease of abnormal Keratinocyte proliferation induced by T lymphocytes. Immunol Today (1986) 7:256–9. 10.1016/0167-5699(86)90005-8 25290627

[37] Ockenfels HM Schultewolter T Ockenfels G Funk R Goos M. The antipsoriatic agent dimethylfumarate immunomodulates T-cell cytokine secretion and inhibits cytokines of the psoriatic cytokine network. Br J Dermatol (1998) 139:390–5. 10.1046/j.1365-2133.1998.02400.x 9767281

[38] Basavaraj KH Ashok NM Rashmi R Praveen TK. The role of drugs in the induction and/or exacerbation of psoriasis. Int J Dermatol (2010) 49:1351–61. 10.1111/j.1365-4632.2010.04570.x 21091671

[39] Bacharach-Buhles M Pawlak FM Matthes U Joshi RK Altmeyer P. Fumaric acid esters (FAEs) suppress CD 15- and ODP 4-positive cells in psoriasis. Acta Derm Venereol Suppl (Stockh) (1994) 186:79–82. 7915483

[40] Rubant SA Ludwig RJ Diehl S Hardt K Kaufmann R Pfeilschifter JM Dimethylfumarate reduces leukocyte rolling in vivo through modulation of adhesion molecule expression. J Invest Dermatol (2008) 128:326–31. 10.1038/sj.jid.5700996 17671516

[41] Garcia-Caballero M Mari-Beffa M Medina MA Quesada AR. Dimethylfumarate inhibits angiogenesis in vitro and in vivo: a possible role for its antipsoriatic effect? J Invest Dermatol (2011) 131:1347–55. 10.1038/jid.2010.416 21289642

[42] Treumer F Zhu K Glaser R Mrowietz U. Dimethylfumarate is a potent inducer of apoptosis in human T cells. J Invest Dermatol (2003) 121:1383–8. 10.1111/j.1523-1747.2003.12605.x 14675187

[43] Lehmann M Risch K Nizze H Lutz J Heemann U Volk HD Fumaric acid esters are potent immunosuppressants: inhibition of acute and chronic rejection in rat kidney transplantation models by methyl hydrogen fumarate. Arch Dermatol Res (2002) 294:399–404. 12522577 10.1007/s00403-002-0347-6

[44] Moed H Stoof TJ Boorsma DM von Blomberg BM Gibbs S Bruynzeel DP Identification of anti-inflammatory drugs according to their capacity to suppress type-1 and type-2 T cell profiles. Clin Exp Allergy (2004) 34:1868–75. 10.1111/j.1365-2222.2004.02124.x 15663561

[45] Hestvik ALK. The double-edged sword of autoimmunity: lessons from multiple sclerosis. Toxins (2010) 2:856–77. 10.3390/toxins2040856 22069614 PMC3153218

[46] Hoglund RA Maghazachi AA. Multiple sclerosis and the role of immune cells. World J Exp Med (2014) 4:27–37. 10.5493/wjem.v4.i3.27 25254187 PMC4172701

[47] Wang Q Chuikov S Taitano S Wu Q Rastogi A Tuck SJ Dimethyl fumarate protects neural stem/progenitor cells and neurons from oxidative damage through Nrf2-ERK1/2 MAPK pathway. Int J Mol Sci (2015) 16:13885–907. 10.3390/ijms160613885 26090715 PMC4490529

[48] Schilling S Goelz S Linker R Luehder F Gold R. Fumaric acid esters are effective in chronic experimental autoimmune encephalomyelitis and suppress macrophage infiltration. Clin Exp Immunol (2006) 145:101–7. 10.1111/j.1365-2249.2006.03094.x 16792679 PMC1942010

[49] Parodi B Rossi S Morando S Cordano C Bragoni A Motta C Fumarates modulate microglia activation through a novel HCAR2 signaling pathway and rescue synaptic dysregulation in inflamed CNS. Acta Neuropathol (2015) 130:279–95. 10.1007/s00401-015-1422-3 25920452 PMC4503882

[50] Giulian D Corpuz M Microglial secretion products and their impact on the nervous system. Adv Neurol (1993) 59:315–20. 8420117

[51] Loewe R Holnthoner W Groger M Pillinger M Gruber F Mechtcheriakova D Dimethylfumarate inhibits TNF-induced nuclear entry of NF-kappa B/p65 in human endothelial cells. J Immunol (2002) 168:4781–7. 10.4049/jimmunol.168.9.4781 11971029

[52] Wierinckx A Breve J Mercier D Schultzberg M Drukarch B Van Dam AM. Detoxication enzyme inducers modify cytokine production in rat mixed glial cells. J Neuroimmunol (2005) 166:132–43. 10.1016/j.jneuroim.2005.05.013 15993952

[53] Asadullah K Schmid H Friedrich M Randow F Volk HD Sterry W Influence of monomethylfumarate on monocytic cytokine formation – explanation for adverse and therapeutic effects in psoriasis? Arch Dermatol Res (1997) 289:623–30. 10.1007/s004030050251 9444385

[54] Vandermeeren M Janssens S Borgers M Geysen J. Dimethylfumarate is an inhibitor of cytokine-induced E-selectin, VCAM-1, and ICAM-1 expression in human endothelial cells. Biochem Biophys Res Commun (1997) 234:19–23. 10.1006/bbrc.1997.6570 9168952

[55] Linker RA Lee DH Stangel M Gold R. Fumarates for the treatment of multiple sclerosis: potential mechanisms of action and clinical studies. Expert Rev Neurother (2008) 8:1683–90. 10.1586/14737175.8.11.1683 18986239

[56] Gold R Linker RA Stangel M. Fumaric acid and its esters: an emerging treatment for multiple sclerosis with antioxidative mechanism of action. Clin Immunol (2012) 142:44–8. 10.1016/j.clim.2011.02.017 21414846

[57] Lee DH Gold R Linker RA. Mechanisms of oxidative damage in multiple sclerosis and neurodegenerative diseases: therapeutic modulation via fumaric acid esters. Int J Mol Sci (2012) 13:11783–803. 10.3390/ijms130911783 23109883 PMC3472775

[58] Scannevin RH Chollate S Jung MY Shackett M Patel H Bista P Fumarates promote cytoprotection of central nervous system cells against oxidative stress via the nuclear factor (erythroid-derived 2)-like 2 pathway. J Pharmacol Exp Ther (2012) 341:274–84. 10.1124/jpet.111.190132 22267202

[59] Itoh K Tong KI Yamamoto M. Molecular mechanism activating Nrf2-Keap1 pathway in regulation of adaptive response to electrophiles. Free Radic Biol Med (2004) 36:1208–13. 10.1016/j.freeradbiomed.2004.02.075 15110385

[60] Li W Kong AN. Molecular mechanisms of Nrf2-mediated antioxidant response. Mol Carcinog (2009) 48:91–104. 10.1002/mc.20465 18618599 PMC2631094

[61] Schimrigk S Brune N Hellwig K Lukas C Bellenberg B Rieks M Oral fumaric acid esters for the treatment of active multiple sclerosis: an open-label, baseline-controlled pilot study. Eur J Neurol (2006) 13:604–10. 10.1111/j.1468-1331.2006.01292.x 16796584

[62] Lull ME Block ML. Microglial activation and chronic neurodegeneration. Neurotherapeutics (2010) 7:354–65. 10.1016/j.nurt.2010.05.014 20880500 PMC2951017

[63] Kunze R Urrutia A Hoffmann A Liu H Helluy X Pham M Dimethyl fumarate attenuates cerebral edema formation by protecting the blood-brain barrier integrity. Exp Neurol (2015) 266:99–111. 10.1016/j.expneurol.2015.02.022 25725349

[64] Haarmann A Nehen M Deiss A Buttmann M Fumaric acid esters do not reduce inflammatory NF-kappaB/p65 nuclear translocation, ICAM-1 expression and T-cell adhesiveness of human brain microvascular endothelial cells. Int J Mol Sci (2015) 16:19086–95. 10.3390/ijms160819086 26287168 PMC4581287

[65] Iniaghe LO Krafft PR Klebe DW Omogbai EK Zhang JH Tang J. Dimethyl fumarate confers neuroprotection by casein kinase 2 phosphorylation of Nrf2 in murine intracerebral hemorrhage. Neurobiol Dis (2015) 82:349–58. 10.1016/j.nbd.2015.07.001 26176793 PMC4640980

[66] Aronowski J Hall CE. New horizons for primary intracerebral hemorrhage treatment: experience from preclinical studies. Neurol Res (2005) 27:268–79. 10.1179/016164105X25225 15845210

[67] Javaid K Rahman A Anwar KN Frey RS Minshall RD Malik AB. Tumor necrosis factor-alpha induces early-onset endothelial adhesivity by protein kinase Czeta-dependent activation of intercellular adhesion molecule-1. Circ Res (2003) 92:1089–97. 10.1161/01.RES.0000072971.88704.CB 12714560

[68] Han R Xiao J Zhai H Hao J. Dimethyl fumarate attenuates experimental autoimmune neuritis through the nuclear factor erythroid-derived 2-related factor 2/hemoxygenase-1 pathway by altering the balance of M1/M2 macrophages. J Neuroinflammation (2016) 13:97. 10.1186/s12974-016-0559-x 27142843 PMC4855950

[69] Lin SX Lisi L Dello RC Polak PE Sharp A Weinberg G The anti-inflammatory effects of dimethyl fumarate in astrocytes involve glutathione and haem oxygenase-1. ASN Neuro (2011) 3:e00055. 10.1042/AN20100033 21382015 PMC3072764

[70] Yogev O Yogev O Singer E Shaulian E Goldberg M Fox TD Fumarase: a mitochondrial metabolic enzyme and a cytosolic/nuclear component of the DNA damage response. PLoS Biol (2010) 8:e1000328. 10.1371/journal.pbio.1000328 20231875 PMC2834712

[71] Frohlich DA McCabe MT Arnold RS Day ML. The role of Nrf2 in increased reactive oxygen species and DNA damage in prostate tumorigenesis. Oncogene (2008) 27:4353–62. 10.1038/onc.2008.79 18372916

[72] Xu C Huang MT Shen G Yuan X Lin W Khor TO Inhibition of 7,12-dimethylbenz(a)anthracene-induced skin tumorigenesis in C57BL/6 mice by sulforaphane is mediated by nuclear factor E2-related factor 2. Cancer Res (2006) 66:8293–6. 10.1158/0008-5472.CAN-06-0300 16912211

[73] Loewe R Valero T Kremling S Pratscher B Kunstfeld R Pehamberger H Dimethylfumarate impairs melanoma growth and metastasis. Cancer Res (2006) 66:11888–96. 10.1158/0008-5472.CAN-06-2397 17178886

[74] Brzozowska I Ptak-Belowska A Pawlik M Pajdo R Drozdowicz D Konturek SJ Mucosal strengthening activity of central and peripheral melatonin in the mechanism of gastric defense. J Physiol Pharmacol (2009) 60(Suppl 7):47–56. 20388945

[75] Herman JP Cullinan WE. Neurocircuitry of stress: central control of the hypothalamo-pituitary-adrenocortical axis. Trends Neurosci (1997) 20:78–84. 10.1016/S0166-2236(96)10069-2 9023876

[76] Shakya A Soni UK Rai G Chatterjee SS Kumar V Gastro-protective and anti-stress efficacies of monomethyl fumarate and a fumaria indica extract in chronically stressed rats. Cell Mol Neurobiol (2015) 36:621–35. 10.1007/s10571-015-0243-1 26215054 PMC11482401

[77] Zhu K Mrowietz U. Enhancement of antibacterial superoxide-anion generation in human monocytes by fumaric acid esters. Arch Dermatol Res (2005) 297:170–6. 10.1007/s00403-005-0598-0 16187092

[78] Yik JH Hu Z Kumari R Christiansen BA Haudenschild DR. Cyclin-dependent kinase 9 inhibition protects cartilage from the catabolic effects of proinflammatory cytokines. Arthritis Rheumatol (2014) 66:1537–46. 10.1002/art.38378 24470357 PMC4127300

[79] Li Y Tang J Hu Y. Dimethyl fumarate protection against collagen II degradation. Biochem Biophys Res Commun (2014) 454:257–61. 10.1016/j.bbrc.2014.10.005 25305493

[80] Oh CJ Kim JY Choi YK Kim HJ Jeong JY Bae KH Dimethylfumarate attenuates renal fibrosis via NF-E2-related factor 2-mediated inhibition of transforming growth factor-beta/Smad signaling. PLoS One (2012) 7:e45870 10.1371/journal.pone.0045870 23056222 PMC3466265

